# Temperature as a Diagnostic and Prognostic Symptom

**Published:** 1888-01

**Authors:** Robert W. Martin

**Affiliations:** Chatham, Va.; Late President of Medical Society of Virginia


### TEMPERATURE AS A DIAGNOSTIC AND PROGNOSTIC SYMPTOM.

By ROBERT W. MARTIN, M. D., CHATHAM, VA.
**Late President of Medical Society of Virginia.**

Human temperature, to be of diagnostic and prognostic value in the study of disease, must be corroborated by other symptoms and the circumstances. While a normal temperature does not necessarily indicate health, a fall below or rise above the natural range shows the individual to be either sick or tending to sickness. Each disease has its fixed laws of temperature and temperature change. To these laws there are exceptions. Of them little is yet known, but by observation our knowledge is increasing; and, already, temperature in many instances affords the best means of diagnosis, and indicates changes, irregularities and tendencies to fatal termination, and often directs the entire course of treatment. In the diseases called typical, such as typhoid fever, lobar-pneumonia, measles, scarlet fever, and the malarial diseases, the temperature affords information so valuable that it is often the chart by which the physician directs his course.

A single observation may be valuable if taken at a significant moment as indicating great danger, or may determine the fact that disease really exists. For example, the extremes of temperature —3° to 5° below and 6° to 10° above the normal—always indicate imminent danger, while a slight febrile temperature may sometimes, as in incipient phthisis and specific fevers, be the only indication of disease.

Some diseases may be diagnosed by the manner of the temperature rise; for example, in pneumonia and other diseases in which the peripheral circulation is diminished and heat given off, producing rigor, the internal temperature rapidly and continuously rises, while typhoid fever, and diseases with a more gradual beginning, show a slow elevation of temperature with an evening rise and morning fall, each day showing slightly higher elevation until the maximum is reached. This may be regarded as one of the most constant unvarying diagnostic symptoms of typhoid fever —the fever mounts every evening 1.5°, and falls back 1°—1.5° every morning for six or eights days, when the maximum of 105° to 106° for the evening, and the minimum of 102° to 104° for the morning temperature is reached. The higher the temperature mounts the greater the danger—106° indicating a decidedly unfavorable prognosis, while 107° or 108° is almost certainly prognostic of a fatal termination.

A higher temperature may be borne in typhoid fever than in pneumonia; in scarlet fever than in measles, and in all diseases a high temperature with perspiration is a graver symptom than when the skin is dry, particularly if the temperature approaches the maximum.

In the beginning of malarial fevers the temperature is habitually high, but speedily returns almost or quite to the normal; therefore, this high temperature, appearing so early, excludes typhoid fever from the diagnosis. In typhoid fever there is sometimes a striking contrast between the elevation of temperature and the frequency of the pulse, the former reaching 103° or 106°, and the latter ranging from 80 to 90 beats per minute. In low temperature, with a frequent pulse, points to chest or pelvic complications, and sometimes to great nervous excitability and prostration, as in the following case:

Mrs ———, aged twenty-seven years, the mother of six children, the youngest fifteen months old, had had several miscarriages, the last one on March 16, 1887. On the 10th of April she was taken with some abdominal pain and considerable uterine hemorrhage, which was promptly relieved. On the 13th she took, of her own accord, a dose of castor oil, which produced excessive purging and brought on a severe and prostrating dysentery; for days the prostration was great and the nervous excitement extreme. The pulse was small and frequent, making 140 beats per minute, but at no time did the thermometer under the tongue indicate a temperature of more than 101°; collapse was due especially to the nervous trouble.

Complications always affect temperature and render both the diagnosis and prognosis more difficult to determine. This is strikingly the case in nervous and hysterical females. Women, who in the beginning of, or immediately after, parturition, exhibit a considerable rise of temperature, must not only be carefully watched, and treated, for eclampsia or some serious kindred trouble is threatened.

In the only case of malarial coma observed, the temperature was, in the beginning, excessively high, but it fell to the normal, and did not rise again even as the coma deepened or death approached.

In order that temperature indications may be valuable frequent observations must be made, always taking into account the time of day, whether before or soon after the taking of food, and, finally, the age of the patient. It may be observed that the nearer the morning temperature keeps to the evening exacerbation, the greater the danger; and, *per contra*, the nearer the morning remission to the normal, the more hopeful is the prognosis. The thermometer and the watch must go together—their joint testimony is much more valuable than that of either alone—*Practice*

